# Resistance in tuberculosis: what do we know and where can we go?

**DOI:** 10.3389/fmicb.2013.00208

**Published:** 2013-07-23

**Authors:** Keith D. Green, Sylvie Garneau-Tsodikova

**Affiliations:** Department of Pharmaceutical Sciences, University of KentuckyLexington, KY, USA

**Keywords:** antibiotics, inhibitors, mechanisms of resistance, new drug targets, synergistic drug therapy

## Abstract

Tuberculosis (TB) has become a worldwide threat, mainly due to the emergence of multidrug-resistant (MDR) and extensively drug-resistant (XDR) strains of *Mycobacterium tuberculosis* (*Mtb*). This mini-review is focused on the various mechanisms of resistance to the currently available anti-TB drugs and provides perspective on novel strategies and lead scaffolds/compounds aimed at inhibiting/overcoming these resistance mechanisms.

## Introduction

**Figure 1:**
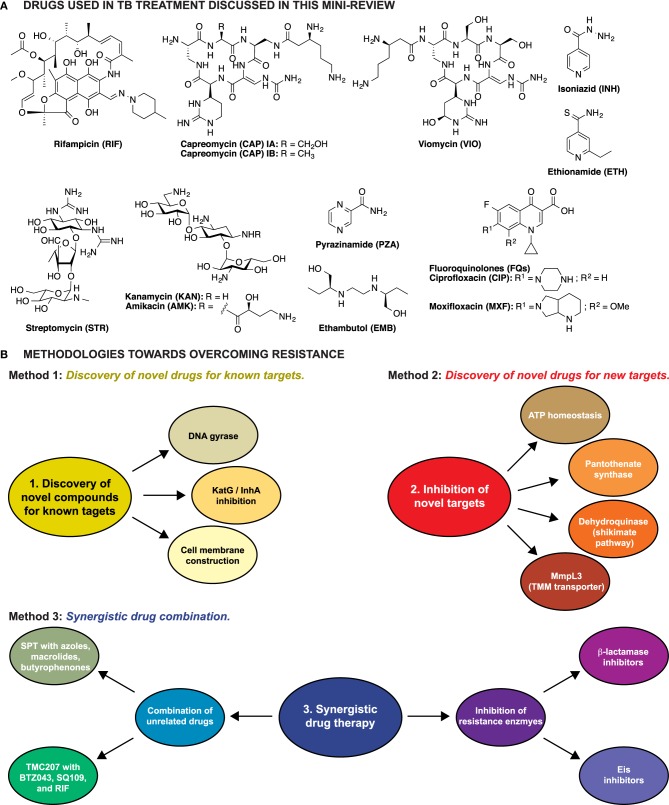
**(A)** Structures of the anti-TB drugs discussed in this mini-review. **(B)** Schematic representation of the three methodologies explored to overcome resistance in *Mtb*.

Approximately 1.4 million deaths were attributed to *Mtb* infections in 2012. When compared to other infectious agents, only HIV claims more lives (WHO, 2012). While recent efforts have resulted in a global decline in TB incidence and mortality (WHO, 2011), the number of individuals infected with drug-resistant isolates continues to increase, presenting a serious global health threat. Generally, drug-susceptible *Mtb* infections are treated with a combination of four compounds; rifampicin (RIF), ethambutol (EMB), pyrazinamide (PZA), and isoniazid (INH) for 2 months, followed by treatment with RIF and INH for 4 additional months (Lienhardt et al., 2012) (Figure 1A). By definition, MDR-TB is resistant to the most potent first-line drugs, RIF and INH. The WHO reports that ~60,000 cases of MDR-TB were diagnosed in 2011, likely an underestimate. To treat MDR-TB, second-line drugs including fluoroquinolones (FQs), amikacin (AMK), kanamycin (KAN), and capreomycin (CAP) are employed. These drugs are administered for ~20 months and can be toxic, poorly tolerated, and difficult to procure. Approximately 95% of MDR-TB is XDR-TB, having additional resistance to at least one FQ and one injectable drug (AMK, KAN, or CAP), and for which treatment options are limited. Therefore, new therapeutics are critically needed to overcome drug resistance and to eliminate TB as a public health threat.

Herein we briefly discuss the drugs currently used to treat TB and the respective mechanisms of resistance followed by a review of approaches aimed toward overcoming TB drug resistance.

## Current drugs and resistance

*Mtb* is intrinsically resistant to many antibiotics due to the low permeability of its mycolic acid-rich waxy cell envelope, the action of efflux pumps (Banerjee et al., 1996; Silva et al., 2001; Singh et al., 2011), and the presence of chromosomally encoded resistance genes. Drug resistance in *Mtb* has been emerging due to the accumulation of chromosomal mutations and not acquisition of mobile genetic elements. The major mechanisms of acquired drug resistance in *Mtb* can be broken down into several categories: (1) mutations or modifications (e.g., RNA methylation) of the drug targets (RIF, EMB, KAN, AMK, CAP, and the FQs), (2) the inability to activate a prodrug [INH, PZA, ethionamide (ETH)] due to mutations leading to a loss of function, and (3) enzymatic inactivation of the drug (KAN).

### Mutations or modifications of the drug targets

#### Mutations

Antibiotics target cellular processes that are vital in bacteria by binding their targets at a specific site, often directly interacting with key functional residues of the target. The most common mechanism of resistance in *Mtb* is the alteration of the target's binding site through the accumulation of mutations. These mutations decrease the binding affinity of the drug to its target and typically occur in a very defined region of the gene termed the resistance-determining region. This mechanism is used by *Mtb* to confer resistance to RIF, EMB, and FQs by altering the binding site of their respective targets: the β-subunit of RNA polymerase (Campbell et al., 2001), a glycosyltransferase (Telenti et al., 1997), and DNA gyrase (Takiff et al., 1994). Similarly, ribosomal mutations (e.g., A1401G) in the 16S rRNA have been found to confer resistance to AMK, KAN, and CAP (Wachino et al., 2010).

#### Modifications

An alternative mode of resistance in *Mtb* is the inactivation of rRNA methyltransferase enzymes. Mutations in TlyA hindering the methylation activity of rRNA 2′-*O*-methyltransferase at nucleotides C1409 of 16S rRNA and C1920 of 23S rRNA have been linked to resistance to the ribosome-targeting drugs CAP and viomycin (VIO) (Johansen et al., 2006). Mutations in a putative 16S rRNA methyltransferase, GidB, were found to confer low-level resistance to the aminoglycoside (AG) streptomycin (STR) (Spies et al., 2011; Wong et al., 2011).

### Inability to activate a prodrug

Three first-line drugs, INH, PZA, and ETH, are prodrugs that must be metabolized to be active against *Mtb*. INH is activated by the catalase-peroxidase, KatG, and produces isonicotinic-acyl radicals that react with NADH to form an INH-NADH adduct. The adduct binds its main target, enoyl-acyl carrier protein reductase (InhA), and inhibits mycolic acid biosynthesis. ETH is a structural analog of INH and is activated by the monooxygenase EthA, which oxidizes ETH to its active form, 2-ethyl-4-amidopyridine. This activated compound also targets InhA and blocks mycolic acid biosynthesis similarly to INH. PZA is activated by pyrazinamidase/nicotinamidase, PncA, to pyrazinoic acid (POA). The proposed targets of POA range from cell membrane energy to fatty acid synthesis, but the exact killing mechanism of POA remains unclear. Resistance to any of the prodrugs can arise due to reduced metabolism by their corresponding activator. Structural mutations that lower or abolish the enzymatic activity of KatG (Ramaswamy et al., 2003; Hazbon et al., 2006), EthA (Baulard et al., 2000; Debarber et al., 2000; Morlock et al., 2003), and PncA (Stoffels et al., 2012; Rajendran and Sethumadhavan, 2013) were found to lead to INH, ETH, and PZA resistance, respectively. Mutations in InhA causing overexpression of the target (Baulard et al., 2000; Debarber et al., 2000; Larsen et al., 2002) or preventing binding of the active form of INH and ETH to the target (Banerjee et al., 1994; Vilcheze et al., 2008) also confer cross-resistance to these drugs.

### Inactivation of the drug

Drug modification is perhaps the most prevalent and well-studied resistance mechanism employed by bacteria. Many bacteria either degrade or modify the offending compound thereby generating an inactive molecule. Resistance to penicillin is mediated by a class of enzymes known as the β-lactamases, which inactivate the antibiotic by destroying the β-lactam ring. *Mtb* naturally harbors a chromosomally encoded class A β-lactamase, BlaC, which is constitutively expressed providing intrinsic resistance to penicillin (Hugonnet and Blanchard, 2007). One mechanism of acquired resistance to AGs, such as KAN and AMK, is their modification and inactivation by a family of enzymes known as AG-modifying enzymes (AMEs) (Ramirez and Tolmasky, 2010; Labby and Garneau-Tsodikova, 2013). *Mtb* expresses two AMEs, the AG 2′-*N*-acetyltransferace AAC(2′)-Ic (Hegde et al., 2001) and the enhanced intracellular survival (Eis) protein (Zaunbrecher et al., 2009). Both enzymes acetylate AGs, reducing the ability of these drugs to inhibit the ribosome. In the case of Eis, increased expression due to mutations in the *eis* promoter or the 5′-untranslated region of the transcriptional activator WhiB7 leads to clinically relevant, low-level resistance to KAN (Zaunbrecher et al., 2009; Reeves et al., 2013). A unique feature of Eis resides in its ability to modify AGs at multiple sites *in vitro*, completely inactivating these compounds (Chen et al., 2011). A recent report has shown that Eis also acetylates CAP at its β-lysine side chain *in vitro*, but the clinical relevance of this finding remains unclear (Houghton et al., 2013).

## Methodologies to overcome resistance

Three major strategies have been explored to overcome resistance in TB: (1) identification of novel drugs for well-established targets, (2) identification and characterization of novel compounds that target unexplored vital cell processes or enzymes, thus killing the cell by preventing regular metabolism, and (3) identification of compounds that behave synergistically with current anti-TB drugs (Figure 1B).

### Discovery of novel drugs for known targets

#### DNA gyrase

Among the known *Mtb* drug targets, DNA gyrase remains attractive for development of inhibitors interacting with either GyrA or GyrB subunits. To overcome the FQ resistance of DNA gyrase mutants, a series of 30 ofloxacin derivatives with various substituents *ortho* to the fluoro moiety of the FQ scaffold were synthesized (Dinakaran et al., 2008). These compounds displayed MIC_99_ values <10 μM and retained anti-mycobacterial activity against MDR-*Mtb* strains. While ofloxacin displayed a 17-fold lower activity against MDR-*Mtb* relative to susceptible *Mtb* strains, its derivatives displayed at most a 4-fold lower activity and, in some cases, higher activity against MDR-*Mtb*. The toxicity of selected compounds in mammalian Vero cells displayed a range of IC_50_ values (60 to >150 μ M), 6-fold higher than the highest MIC_99_ value. When tested in culture, the compound with the best MIC_99_ value showed reduction in *Mtb* colonies similar to that observed with ofloxacin.

While many efforts have been directed toward the development of FQ analogs to target the GyrA subunit of DNA gyrase, development of inhibitors targeting GyrB has also been pursued. A series of aminopyrazinamides were found to target the ATPase site of GyrB and inhibit the growth of *Mtb* in the low micromolar/high nanomolar range (Shirude et al., 2013). A variety of naphthoquinones were also discovered to inhibit the ATPase domain of the GyrB subunit of *Mtb* DNA gyrase with IC_50_ values between 15 and >200 μ M (Karkare et al., 2013). The most promising naphthoquinone, diospyrin, was tested against DNA gyrase from other bacterial species and found to be a broad spectrum DNA gyrase inhibitor. The aminobenzimidazole AB-1 was also investigated for its inhibitory properties of GyrB and compared to the well-characterized GyrB inhibitor novobiocin (Chopra et al., 2012). AB-1 displayed lower MIC values than novobiocin with *Mtb* strains resistant to STR, INH, CIP, and *p*-aminosalicylic acid. AB-1 also displayed improved activity in aerobic and anaerobic conditions while not interfering with RIF or INH mechanisms.

#### KatG/InhA inhibition

To overcome mutations of KatG and other mechanisms of INH resistance, analogs of INH have been synthesized. Early generations of INH analogs incorporated aryloxyacetonitriles onto the hydrazide of the INH scaffold (Bukowski et al., 1999). These compounds were then evolved into 1,2,3-triazole derivatives with MIC values slightly higher than those of INH (Boechat et al., 2011).

As an alternative strategy to overcoming KatG mutations or deletion, InhA inhibitors, not needing KatG activation, have been investigated. Triclosan (Parikh et al., 2000) served as a lead compound for the design of a series of 5-alkyl-diphenyl ethers (Sullivan et al., 2006). These ethers displayed IC_50_ values for InhA ranging from 5 nM to 2 μ M, displayed higher bactericidal activity than the parent triclosan, and were further optimized to slow the release time from the InhA active site (Luckner et al., 2010). More recently, from screening 300 molecules, CD117 was identified as an InhA inhibitor that likely targets other enzymes (Vilcheze et al., 2011). CD117 was active against hypoxic *Mtb* and yielded 80% Vero cell viability at the highest concentration tested. This year, virtual screening efforts led to the identification of 7 potential InhA inhibitors (Kinjo et al., 2013). In model *Mycobacterium* species the top hit compounds displayed similar growth inhibition to that of INH and directly inhibited *Mtb* InhA.

#### Cell membrane construction

The inhibition of cell membrane construction also remains a target of choice for the development of new drugs. Nitroimidazoles such as PA-824 and OPC-67683 have been found to inhibit the synthesis of membrane proteins and lipids (Mukherjee and Boshoff, 2011). Much like INH and PZA, PA-824 is a prodrug that requires activation by reduction of its nitro group (Stover et al., 2000). PA-824 and OPC-67683 have demonstrated activity against both susceptible and resistant-*Mtb* strains (Stover et al., 2000; Rivers and Mancera, 2008a,b). PA-824 showed activity in the high ng/mL range for both susceptible and resistant-*Mtb* (Ginsberg et al., 2009) and OPC-67683 displayed better MIC values in the low ng/mL range (Matsumoto et al., 2006).

Benzothiazinones (BTZs) (Makarov et al., 2006), such as BTZ043, have also been shown to interfere with cell membrane construction. Like EMB, these compounds disrupt arabinan's biosynthesis by targeting decaprenylphosphoryl-β-d-ribose 2′-epimerase (DprE) (Makarov et al., 2009). In infected macrophages, BTZ043 showed a faster decrease in percentage of infected cells and bacterial load compared to INH or RIF. BTZ043 has also been tested in 240 clinical isolates of *Mtb* from European hospitals and was active against all strains (Pasca et al., 2010).

### Discovery of novel drugs for new targets

#### ATP homeostasis

One of the challenges in treating TB is the organism's extended period(s) of dormancy. One key difference in the states of growing and hypoxic non-growing *Mtb* is a reduced, but significant, ATP pool in the non-growing organism (Rao et al., 2008). A plausible way to attack the non-growing bacilli is to affect their ATP storage by inactivating ATP synthase to deplete the ATP and eventually kill the bacilli (Andries et al., 2005). This strategy led to the identification of the ATP synthase inhibitor TMC207 (bedaquiline or sirturo), which was approved by the FDA in December 2012 for TB treatment (Diacon et al., 2009; Avorn, 2013). TMC207 is the first FDA-approved anti-TB drug in 40 years (Mahajan, 2013). TMC207 targets the C-subunit of ATP synthase proton pump and mutations are known that are resistant to this novel compound (Segala et al., 2012). There are also some mycobacterial strains that are naturally resistant to TMC207. Because of the potential risks associated with this drug, it is recommended that it be utilized only in patients for which other treatment options have failed. A high-throughput screen (HTS) to identify additional compounds that block ATP synthesis and kill dormant *Mtb* resulted in 0.5% hit rate from a library of 600,000 compounds (Mak et al., 2012).

#### Pantothenate synthetase

Pantothenate synthetase has also been explored as an antibiotic target of *Mtb*. Actinomycin D was identified *via* HTS as an inhibitor of this *de novo* synthesis enzyme (Yang et al., 2011). Futher *in silico* HTS led to the discovery of two compounds that inhibit the panthothenate synthesis 10× more efficiently than actinomycin D does. These compounds displayed identical MIC values against susceptible and MDR-*Mtb* strains. Analogs of the pantothenate synthetase reaction intermediate were shown to bind the enzyme more tightly than the nucleotide substrate (Ciulli et al., 2008). Another essential enzyme, pantothenate kinase (CoaA) has also been considered as a target. Three classes of compounds were found to target CoaA: triazoles, quinoline carboxamides, and biaryl acetic acids (Venkatraman et al., 2012). All compounds inhibited CoaA with IC_50_ values ranging from 70 nM to 8.4 μ M with various modes of inhibition.

#### Dehydroquinase of the shikimate pathway

The essential shikimate pathway, responsible for the biosynthesis of aromatic amino acids, folate, and vitamins E/K, is also an antibacterial target (Gonzalez-Bello and Castedo, 2007; Payne et al., 2007a,b; Tran et al., 2011). Recently, inhibitors of the dehydroquinase enzyme of the shikimate pathway have been optimized against *Mtb* and a series of 3,4-dihydroxyacetophenone and acetonide cores with MIC values in the low-micromolar range were identified (Tran et al., 2012). Similarly, a series of anhydroquinones were synthesized and found to be low-micromolar to high-nanomolar *Mtb* dehydroquinase inhibitors (Payne et al., 2007a; Prazeres et al., 2007). Other studies have focused on generating dehydroquinic acid derivatives (Lence et al., 2013) or 3-alkyl enol mimics (Blanco et al., 2012) to inhibit dehydroquinase and disrupt the shikimate pathway. Dehydroquinic acid analogs showed IC_50_ values in the mid-nanomolar range (26–100 nM), while the 3-alkyl enol mimics had a broader nanomolar range (28–780 nM).

#### MmpL3 (TMM transporter)

SQ109 is an EMB analog with an adamantyl moiety that targets cell well-biosynthesis. SQ109 was discovered in a screen of ~63,000 compounds and showed high ng/mL MIC values (Sacksteder et al., 2012). Recent studies revealed that SQ109 acts on MmpL3, the trehalose monomycolate (TMM) transporter of *Mtb* (Tahlan et al., 2012). Adamantyl ureas, discovered by high-throughput screening, also displayed anti-tubercular activity (Brown et al., 2011) by targeting MmpL3 (Grzegorzewicz et al., 2012). Since their discovery, the structure of organic ureas has undergone several optimizations for solubility (Scherman et al., 2012) and pharmacokinetic properties (North et al., 2013). Additional scaffolds based on the structure of the pyrrole derivative BM212 have also recently emerged as MmpL3 inhibitors (Poce et al., 2013).

### Synergistic drug combination

#### Combination of unrelated drugs

In an effort to reduce emerging resistance, combinations of small molecules have been explored. In a search for compounds that would work synergistically with spectinomycin (SPT), a high-throughput synergy screen (HTSS) was performed with a library of molecules with known pharmacological properties. Three structural cores were found to enhance the antibiotic activity of SPT: the macrolides, azoles, and butyrophenones (Ramon-Garcia et al., 2011). Bromperidol displayed bactericidal activity, was effective against *Mtb* in a macrophage model, and displayed similar enhancement of the activities of RIF, STR, clofazimine, and clarithromycin. In addition to its anti-TB activity, TMC207 has also been shown to speed up treatment with the first-line anti-TB drugs (Diacon et al., 2009). Lechartier et al. combined BTZ043 with known and experimental TB treatments (Lechartier et al., 2012). In most cases additive interactions of BTZ043 with the second TB treatment were observed. However, BTZ043 was shown to act synergistically with TMC207. Both drugs used at one-quarter of the MIC concentration showed more significant growth retardation than TMC207 at its MIC value alone. The authors suggest that BTZ043 inhibits DprE1 leading to weaker cell walls allowing transmission of TMC207 across the cell membrane. In a recent study, SQ109 acted synergistically with RIF and TMC207 (Reddy et al., 2010).

#### Inhibition of resistance enzymes

The use of inhibitors of drug-modifying enzymes along with currently approved anti-TB drugs complements the drug combination strategy described above. Perhaps the best-known combination of this type is a β-lactam and a β-lactamase inhibitor. A combination of clavulanate and meropenem proved to be active against both susceptible and XDR-*Mtb* strains (Hugonnet et al., 2009) A novel β-lactamase inhibitor, NXL104, was recently reported and found to bind BlaC in a 1:1 molar ratio (Xu et al., 2012). Recently, inhibitors of the AG-resistance enzyme Eis were discovered (Green et al., 2012). These compounds encompass a wide variety of chemical structures and offer a plethora of scaffolds to be studied for development of novel anti-TB drug adjuvants. While these compounds have not yet been tested in AG-resistant *Mtb* strains, combinations of inhibitors with AGs are predicted to restore the antibacterial activity of AGs in cells.

## Conclusion

Even though current TB treatments are still broadly employed, the rapid spread of MDR-TB and XDR-TB is a rising threat. Based on our current understanding of the resistance mechanisms, contemporary strategies are focused on generating compounds that will avoid or overcome the defenses of *Mtb*. Research is being carried out to find novel compounds that will disrupt DNA, cell membrane biosynthesis, and general cellular metabolism. Other lines of inquiry are focusing on pathways currently not used to treat TB: cellular energetics, cellular transport, and other aspects of cellular metabolism. A third facet relies on current TB treatment and combines drugs to achieve synergistic effects, either blocking two separate pathways or inhibiting a drug-modifying enzyme. While many of the compounds discussed herein are far from being approved as drugs, the recent approval of TMC207 and the multitude of efforts in searching for novel antibiotics give hope for the discovery of novel TB treatments.

### Conflict of interest statement

The authors declare that the research was conducted in the absence of any commercial or financial relationships that could be construed as a potential conflict of interest.
